# Population-based epidemiology of non-fatal injuries in Tehran, Iran

**DOI:** 10.15171/hpp.2018.16

**Published:** 2018-04-18

**Authors:** Esmatolsadat Hashemi, Mahdi Zangi, Homayoun Sadeghi-Bazargani, Joaquim Soares, Eija Viitasara, Reza Mohammadi

**Affiliations:** ^1^Department of Health Sciences, Unit for Public Health Science, Mid Sweden University, Sundsvall, Sweden; ^2^Tracheal Diseases Research Center, Shahid Beheshti University of Medical Sciences, Tehran, Iran; ^3^Road Traffic Injury Research Center, Department of Statistics & Epidemiology, Tabriz University of Medical Sciences, Tabriz, Iran; ^4^Department of Neurobiology, Care Sciences and Society, Unit for Family Medicine, Karolinska Institute, Stockholm, Sweden

**Keywords:** Injuries, Accidents, Epidemiology, Road traffic injuries, Burns, Falls, Iran, Tehran

## Abstract

**Background:** Our aim in this survey was to explore descriptive epidemiology of injuries in Tehran in 2012 and to report the recalled estimates of injury incidence rates.

**Methods:** A population survey was conducted in Tehran during 2012, within which a total of 8626 participants were enrolled. The cluster sampling was used to draw samples in 100 clusters with a pre-specified cluster size of 25 households per cluster. Data were collected on demographic features, accident and injury characteristics based on the International Classification of Diseases (ICD10).

**Results:** A total of 618 injuries per 3 months were reported, within which 597 cases (96.6%)were unintentional injuries. More than 82% of all injuries were those caused by exposure to inanimate mechanical forces, traffic accidents, falls and burns. Above 80% of the traffic injuries happened among men (P<0.001). About 43% of the unintentional injuries were mild injuries.After the age of 40, women, unlike men, had higher risks for being injured. The estimated annual incidence rate for all types of injuries was 284.8 per 1000 (95% CI: 275.4-294.4) and for unintentional injuries was 275.2 per 1000.

**Conclusion:** Injuries are major health problems in Tehran with a highly reported incidence. The status is not substantially improved over the recent years which urges the need to be adequately and emergently addressed. As the incidence rate was estimated based on participant recalls, the real incidence rate may even be higher than those reported in the current study.

## Introduction

Among the most challenging problems ahead of health policy makers and political leaders is decreasing the burden of injuries. Injuries often result in either death or a major deterioration of quality of life among the survivors. A majority of this burden belongs to low and middle income countries (LMICs), despite its high priority for high income countries.^1,2^

Compared to other injuries, road traffic injuries (RTIs) are of higher importance in Iran. In this country, after ischemic heart diseases (9.1%) and low back pain (9%), RTIs (7.3%) are considered as the third major contributor to disability adjusted life years (DALYs). In Iran, similar to the global status, RTIs in both genders are the first cause of mortality among 15-49 years old population.^3,4^

Regardless to the large similarity of LMICs in injury burden, the magnitude and epidemiological characteristics of injuries in LMICs are difficult to establish which may be due to the lack of valid and representative data.^5,6^ A majority of literature in the field of injuries in Iran is originated from hospital based studies. In order to capture the overall epidemiologic pattern of injuries there is a need for repeated population-based studies which are rarely available in Iran and many LMICs.

Tehran is the capital of Iran with a population more than 8.8 million. Tehran is the third largest city in the Middle East, after Cairo and Istanbul. The most recent population-based study in Tehran was conducted in 2008.^7^ This study seemed to be the most recent comprehensive population-based general injury survey prior to the present study. Although several injury specific surveys have been conducted including a much recent work by the same authors.^8^

Tehran municipality started Tehran safe community program to evaluate the impact of its activities, to find a baseline survey and to map out the epidemiology of injuries in Tehran metropolitan.

There is an urgent need for investigating the effectiveness of community-based interventions with the hope to conduct a baseline assessment of the prevalence of various injuries before interventions. Such assessments have not been seriously considered in safe community interventions in many LMICs and Iran,^9,10^ as well. As, before the safe community movement in capital of Iran, no comprehensive study was found in the literature to depict the epidemiological pattern of injuries in Tehran, the current survey was conducted to explore descriptive epidemiology of injuries in Tehran in 2012 and to report the recalled estimates of injury incidence rates.

## Materials and Methods

### Study population and sampling

This cross-sectional population survey was conducted in Tehran, during 6 months in 2012. The households who were willing to participate in the study were considered eligible to be enrolled in the survey.

Cluster sampling was used to draw samples in 100 clusters of the households in Tehran District with a pre-specified 25 households per cluster. Considering an average family size of 3.4 per household in Tehran, a total of 8500 samples were assumed to be recruited. The sample size was estimated based on: injury occurrence =3%, acceptable accuracy = 0.5%, the confidence level = 95%, design effect = 1.8 and a potential attrition rate = 5%. Clusters were selected based on a probability proportional to size (PPS) using the national census 2011 database. The addresses for the selected households were extracted. After determining the start point to initiate the survey in each cluster, consecutive households were enrolled following the right-hand rule to capture 25 residential locations. No institutional participants were enrolled.

### Data collection

Data collection was conducted by trained interviewers most of whom had a bachelor degree. Prior to the beginning of survey, the interviewers participated in a training session to learn about the research plan and their duties throughout the survey. The survey process was well described and the interviewers were supplied with the data collection package and the printed maps of the addresses. In the session, the data collection tools were reviewed in detail and all possible questions were answered to remove ambiguities. Besides, they were provided with a 12-page guideline providing full description of the questionnaires for their potential future referrals. A supervisory team was also trained who participated both in the training session for the interviewers and in a separate training session for monitoring the data collection process. Then, a pilot interview was planned in a test location through which each interviewer interviewed two households. A day later, they came back to a discussion room where they described their experiences and raised possible questions they might had. This was planned to reduce the interrater variability of the assessments.

A questionnaire for interview was developed, evaluated and improved to be used as data collection tool in this survey. This data collection tool was developed to collect the survey data elements recommended by the World Health Organization (WHO) guidelines for conducting community surveys on injuries and violence. This questionnaire was developed according to the WHO framework for conducting community safety surveys.

The main data collected in this survey included demographic data, accident occurrence data and injury-related data such as injury mechanism, injured body organ, activity type while injured and injury place. Injury-related data were collected based on the classifications determined in the International Classification of Diseases (ICD10). The socioeconomic status was assessed as applying a valid and reliable instrument called the SESIran socioeconomic status tool.^11,12^ The total direct costs for medical care received by the injured participants were also recorded (in Iranian Rials) based on the patients’ self-reports. Data were analyzed using Stata version 13 statistical software package. Descriptive statistics such as frequencies and relative frequencies as well as means and standard deviations were reported.

## Results

**Figure 1:**
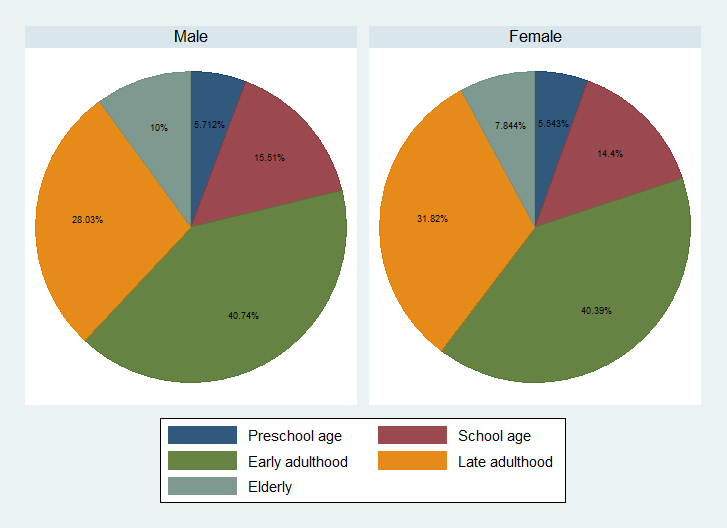
The age distribution among males and females participating in Tehran injury survey.

In the present study, a total of 2498 households out of 2500 pre-specified cases were investigated (response rate = 99%). A total of 8626 household members were surveyed. The sample included 4359 (50.6%) male and 4260 (49.4%) female participants. Children comprised 5.7% of the subjects followed by older adults, forming 8.9% of all enrolled participants. Figure 1 presents age distribution among male and female respondents.

**Figure 2:**
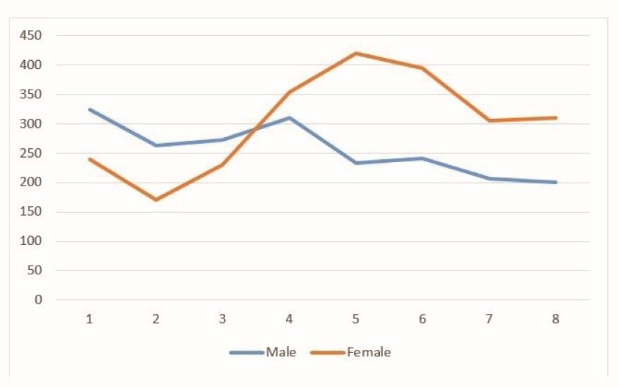
The annual generalized incidence rates of injuries of any type for various sex-age groups in Tehran 2012.

A total of 618 injuries per 3 months were reported, within which 597 (96.6%) cases were unintentional injuries. The annual generalized incidence rates for various sex-age groups are shown in Figure 2. After the age of 40, compared to other age groups, the women had higher risks to be injured and this risk was the highest after the age of 50. This trend, however, was slightly decreasing among men. The estimated annual incidence rate for all type of injuries was 284.8 per 1000 (95% CI: 275.4-294.4). The estimated annual incidence rate for unintentional injuries was 275.2 per 1000 (95% CI: 265.8-284.6).

More than 82% of the injuries were caused by exposure to inanimate mechanical forces followed by traffic accidents, falls and burns. A significant difference was observed in injury mechanisms by gender, in a way that above 80% of the traffic injuries were happened among men (*P *< 0.001) (Table 1).

**Figure 3:**
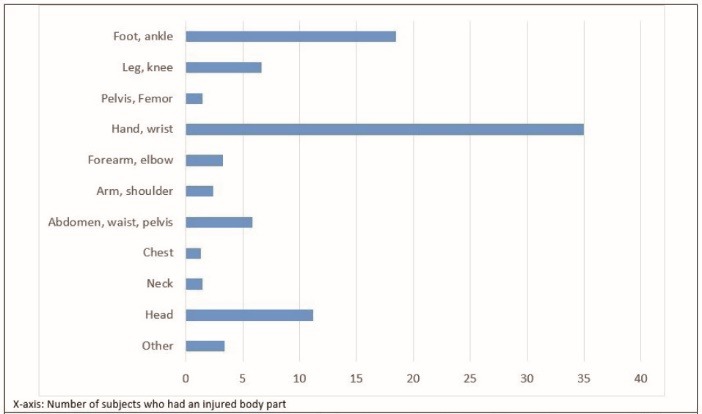
Distribution of injuries according to the body organ involved in Tehran injury survey 2012.

Among unintentional injuries, the mild injuries comprised 43% of the injuries followed by burns (14%), fractures (13%), open wounds (11.6%), and sprains, strains and subluxations (7.2%). Upper and lower limbs were the most common organs involved in the injuries (Figure 3).

The accident economic loss was reported in Iranian Rials (IRRs). Mean and median costs were 13 622 030 and 3 500 000 IRRs, respectively. Mean medical costs of the injuries was 8 605 767 IRRs with a median cost of 1 000 000 IRRs (SD = 2 670 8870 IRRs, IQR = 3 500 000 IRRs). The most common place of injuries in nearly half of the accidents was home, followed by roads (30%). Types of activities distribution at the time of accidents and the accidents’ place are illustrated in Table 2.

About one-third (213) of the injured participants had received medical care for their injury. During the first medical referral, 138 (64.5%) cases referred to hospital, 27 (12.6%) cases referred to medical clinics, 25 (11.7%) cases referred to public health centers, 17 (7.9%) cases referred to general practitioners and the remaining referred to the paces other than the formal health system, such as traditional care givers, bonesetters and pharmacists. About 105 of the injured participants had a second medical referral, within which 94% referred to hospitals, medical clinics and general practitioners. Forty-three subjects had a third referral, among which 80% had hospital referrals.

The current life activities (such as attending work, school and recreation activities) of 26.9% of the individuals were affected by the injury. The injury led to losing job in 3 (1.4%) subjects who were employed prior to their accident. Among 223 injured participants who were employed or were students, 59 (26.5%) cases had at least one working day lost from their work or university. Mean and median days lost were 16.1 and 7 days, respectively (SD = 22.9: IQR = 17). The injury had caused total disability in one case and four other cases had permanent single organ disability. Only one mortality was reported who was a 71-year-old man died in hospital after a traffic accident.

## Discussion

A major portion of injuries in our study comprised the minor injuries. This is quite reasonable due to the sensitivity of population-based surveys in mapping the epidemiology of injuries, even for minor injuries. In a prior similar study conducted in Tehran in 2007 to 2008, similar proportion was observed for minor injuries.^7,13^ However, the rate of minor injuries had a higher proportion in the present study compared to those mentioned in previous studies. In the present study in order to reduce the possibility of recall bias for minor injuries, we investigated the recall for injuries over 3-month periods rather than the 12-month recall as conducted in the earlier studies. The best estimation, however, could be provided only in prospective data collection strategies. A previous study on burn injuries with a prospective design over a 12-month period showed that minor burns comprised 95% of all burns,^14^ which was in contrast to those found in the studies conducted in clinical settings and even cross-sectional population surveys. Although minor injuries have a lower level of importance in terms of health outcome, the risk situation causing a minor injury may not be very different from that in a severe injury, in many instances.

In the present study, the incidence of injury in Tehran was higher than the estimates reported earlier. More than 82% of all injuries were due to four main mechanisms: exposure to inanimate mechanical forces, traffic accidents, falls and burns. Although the overall pattern of the injuries were similar to those reported in 2008, in our study exposure to inanimate mechanical forces were the leading cause of injuries which is contrary to the finding reported in 2008, within which RTIs were found to be the most common type of injury. Regardless the possibility of change in the patterns of injury occurrence, it is supposed that the difference between the findings in our study and those reported in 2008 may be due to the higher sensitivity of current study in detecting minor injuries, which may have a different pattern from severe injuries. Similarly, when comparing the incidence rates, the higher injury rates in the present study was mainly explained by higher sensitivity of current study in detecting minor injuries using a 3-month recall versus 12-month recall in previous studies which may claim bias towards underestimating the minor injuries. However, a less likely assumption may be the increased rate of injury incidence which is not in line with the available evidence.^15^

Age has always been a matter of interest in injury research such that some age groups contribute to majority of the injury burden. For example traffic injuries were reported to be the leading cause of death in 15-29 years age group.^16-18^ Moreover, the largest absolute increases in the global total rate of years of life with disabilities (YLDs) were shown to lie between the ages of 40 and 69.^19^

In present study, women after the age of 40 were at higher risks of being injured. This risk was the highest at the 50th decade of their age. This trend however was slightly decreasing among men. In a population survey in Germany, it was similarly found that injury incidence rate in the adulthood was sharply decreasing by age among men but this rate for women was constant by their age. The difference in the population pyramids should be taken into account when comparing the rates not standardized to a reference population.^20^ Saadat et al also found a similar pattern for the injury incidence rate by age for men. In their study, a very sharp increase was reported in the injury incidence rate at the seventh decade of age which could mostly be attributed to the imprecision, because only 11 people were injured in this age group with a substantially wide confidence interval of annual incidence rate.^7^ In an Italian study that was based on emergency department data, both genders had similar distributions in injury incidence rate up to 41-50 years of age, followed by an increasing rate among women and a decreasing rate among men at the higher ages.^21^ Although, the lower rates reported in high income countries may be explained by the real lower rates than those reported in Iran, as a developing country, we believe in higher sensitivity of detecting injuries through shorter time recall period in Tehran, except for the traffic injuries which is a real catastrophic event in Iran.^15,20-26^

In the present study, the injuries caused by exposure to inanimate mechanical forces followed by traffic accidents, falls and burns comprised more than 82% of all injuries. Home was the most common place of injury, except for RTIs which are intrinsically known to be occurred in traffic environment. This pattern was similar to many previous studies.^7,8,27,28^ Home has the fame to be a safe haven by most people. However, it can be a dangerous place where injuries are frequently occurred. Different injury prevention programs may be needed at homes which should be closely in accordance with evidence to improve the efficiency of home safety promotion programs.^5,6^

The present study was a population survey and showed that the incidence of injury in Tehran was higher than the estimates driven from hospital-based studies. Contrary to the hospital-based studies, our study was conducted to assess the incidence rates of injuries regardless of their fatality or severity.^29^ Because not all the injuries are admitted to hospitals, and some cases are referred to peripheral clinics and some cases even do not seek for medical services.

We concluded that injuries are major health problems in Tehran with a highly reported incidence rate that is not substantially improved over the recent years when compared to the latest reliable findings. As the incidence rate in the present study was based on participant recalls, the real incidence may even be more than those reported in the current study. Although the sudden nature of injuries makes it possible to assess the incidence of injuries through recall citations while conducting cross-sectional population-based surveys, this is always subject to underestimation of the incidence rate due to recall limitations. Such a limitation is not an exception to the current study. In order to reduce this effect in the present study, the recall period was restricted to three months. Nevertheless, it should be taken into account that the real incidence rate could even be higher than the observed incidence. As current study was not specifically developed for economic assessment purposes and the recorded costs were just based on self-reports, the results related to injury costs should be cautiously interpreted, especially taking into account that the potential underestimation of costs are more likely to occur.

## Ethical approval

The research protocol was approved at the organizational committee of ethics in Tabriz University of Medical Sciences (Ethics No. 817/4/5 dated on April 20, 2011). Written informed consent was obtained from all the participants.

## Competing interests

The authors declare that they have no competing interests.

## Authors’ contributions

All authors were involved in drafting the article or revising it critically for important intellectual content, and all authors approved the final version to be submitted for publication. Acquisition of data was done by MZ and EH. RM, EV, JS and HSB supervised the whole research process. HSB & RM had full access to all of the data in the study and supervised the data analysis conducted by PhD student EH and MZ. All authors participated in study conception and design as well as interpretations of results.

## Acknowledgments

The authors are thankful to Tehran Municipality for financially supporting the study and Iranian International Safe Community Support Center (Tabriz) for their scientific support. Declaration: This work was conducted as part of a PhD thesis project in Mid Sweden University, Sundsvall, Sweden.

**Table 1:** Distribution of injury mechanisms by gender in Tehran 2012

	**Male** **No. (%)**	**Female** **No. (%)**	**Total**
Traffic accident	109 (81.3)	25 (18.7)	134
Fall	46 (35.7)	83 (64.3)	129
Exposure to inanimate mechanical forces	66 (38.8)	104 (61.2)	170
Bites & animal attack	18 (58.1)	13 (41.9)	31
Burns	11 (13.4)	71 (86.6)	82
Other	25 (54.4)	21 (45.7)	46
Missing or unknown	6 (60)	4 (40)	10
Homicide & suicide	6 (42.9)	8 (57.1)	14
Total	287 (46.6)	329 (53.4)	616

**Table 2:** Types of activities at the time of accidents and the accidents’ place in Tehran injury survey, 2012

**Variable measured**	**Variable labels**	**Frequency**	**Percentage**	**95% CI for percentages**
Accident place				
	Home	307	49.68	45.7-53.6
	School and educational centers	33	5.34	3.8-7.4
	Sport locations	12	1.94	1.1-3.4
	Roads	183	29.61	26.1-33.3
	Industrial or construction sites	20	3.24	2.1-5
	Recreation places	16	2.59	1.6-4.2
	Other	27	4.37	3-6.3
	Missing or unknown	20	3.24	2.1-5
Type of activity while injured				
	Sport activities	28	4.85	3.4-6.9
	Recreation	63	10.92	8.6-13.7
	Occupational with income	89	15.42	12.7-18.6
	Other work-related activities	99	17.16	14.3-20.5
	Eating, resting, home chores	277	48.01	43.9-52.1
	Others	21	3.64	2.4-5.5
